# Efficacy of bisphosphonate therapy on postmenopausal osteoporotic women with and without diabetes: a prospective trial

**DOI:** 10.1186/s12902-022-01010-w

**Published:** 2022-04-11

**Authors:** Jinyoung Kim, Kyoung Min Kim, Soo Lim, Moo-Il Kang, Ki-Hyun Baek, Yong-Ki Min

**Affiliations:** 1 Division of Endocrinology and Metabolism, Department of Internal Medicine, Yeouido St. Mary’s Hospital, The Catholic University of Korea College of Medicine, 10 63-ro Yeongdengpo-gu, Seoul, 07345, Korea; 2 Division of Endocrinology and Metabolism, Department of Internal Medicine, Yongin Severance Hospital, Yonsei University College of Medicine, 363 Dongbaekjukjeon-daero Giheung-gu, Yongin-si, Gyeonggi-do 16995 Korea; 3 Division of Endocrinology and Metabolism, Department of Internal Medicine, Seoul National University Bundang Hospital, Seoul National University College of Medicine, 82 Gumi-ro 173 Beon-gil Bundang-gu, Seongnam-si, Gyeonggi-do 13620, Korea; 4 Division of Endocrinology and Metabolism, Department of Internal Medicine, Seoul St. Mary’s Hospital, The Catholic University of Korea College of Medicine, 222 Banpo-daero Seocho-gu, Seoul, 06591 Korea; 5 Division of Endocrinology and Metabolism, Department of Internal Medicine, Samsung Medical Center, Sungkyunkwan University School of Medicine, 81 Irwon-Ro Gangnam-gu, Seoul, 06351 Korea

**Keywords:** Type 2 diabetes mellitus, Osteoporosis, Bisphosphonates

## Abstract

**Background:**

The co-occurrence of diabetes and osteoporosis is common in postmenopausal women. For the treatment of postmenopausal osteoporosis, current guidelines recommend initial treatment with bisphosphonates, but it is unclear whether bisphosphonates provide a similar degree of therapeutic efficacy in patients with diabetes. This study sought to compare the efficacy of monthly oral ibandronate for retaining bone mineral density (BMD) in diabetic and non-diabetic postmenopausal women with osteoporosis.

**Methods:**

Postmenopausal osteoporotic women with or without diabetes were enrolled in this study from three hospitals in an open-label approach from 2018 to 2020. Each group of patients received oral ibandronate 150 mg once monthly for 1 year. BMD, trabecular bone score (TBS), serum C-terminal telopeptide of type I collagen (CTx) and procollagen type 1 N-terminal propeptide (P1NP) were evaluated prospectively. Treatment-emergent adverse events and changes in glucose metabolism during drug use were also monitored.

**Results:**

Among the 120 study participants, 104 (86.7%) completed the study. Following 1 year of treatment, BMD increased by 3.41% vs. 3.71% in the lumbar spine, 1.30% vs. 1.18% in the femur neck, and 1.51% vs. 1.58% in the total hip in the non-diabetes and diabetes groups, respectively. There were no significant differences in BMD changes between the groups, and the differences in CTx or P1NP changes between groups were not significant. We did not observe any significant differences in baseline TBS values or the degree of change between before and after 1 year of ibandronate treatment in either group in this study. A total of 11 adverse events (9.2%) that recovered without sequelae occurred among the 120 included patients, and there was no significant difference in the frequency of adverse events between the groups (*p* = 0.862). The changes in fasting glucose and glycated hemoglobin levels between before and after treatment were not significant in the diabetic group.

**Conclusions:**

Bisphosphonate therapy showed similar increases in BMD and decreases in CTx and P1NP of postmenopausal women with and without diabetes. Monthly oral ibandronate can be a safe and effective therapeutic option in postmenopausal osteoporosis patients with type 2 diabetes.

**Trial registration:**

NCT number: NCT05266261, Date of registration: 04 March 2022.

**Supplementary Information:**

The online version contains supplementary material available at 10.1186/s12902-022-01010-w.

## Background

Type 2 diabetes mellitus (T2DM) and osteoporosis are common in postmenopausal women [1]. Previous studies have reported that diabetes can cause deterioration of bone quality due to accumulation of advanced glycosylation end products in bone architecture or progressive microangiopathy surrounding the bone environment [2–5]. In addition, the risk of falls increases due to the occurrence of hypoglycemic events [6] and the diabetic complications such as diabetic retinopathy [7], diabetic peripheral neuropathy [8], and diabetic autonomic neuropathy. Therefore, more attention is needed to treat osteoporosis and prevent fractures in diabetic patients [9].

In postmenopausal women at high risk of fracture, current guidelines recommend initial treatment with bisphosphonates including alendronate, risedronate, zoledronic acid, and ibandronate to reduce fracture risk [10, 11]. However, it is unclear whether bisphosphonates provide a similar degree of therapeutic efficacy in patients with diabetes, who can present with complex situations; such as poor bone quality, multiple comorbidities, or use of various medications. Bisphosphonates reduce osteocalcin level by inhibiting osteoclast activity [12], and osteocalcin is associated with insulin secretion and sensitivity [13]. Not surprisingly, concerns have been raised that bisphosphonate therapy can adversely affect glucose metabolism [14, 15].

However, few studies have investigated the efficacy and safety of ibandronate in patients with T2DM. Ibandronate is a therapeutic agent that can be applied with intermittent dosing because it is sequestered in the bone with high binding affinity [16]. Once-monthly oral ibandronate, in a 150 mg tablet, has been deemed effective for increasing bone mineral density (BMD) and preventing osteoporotic fractures [17, 18]. Although the effectiveness of ibandronate for preventing non-vertebral fractures is controversial, it is used widely due to the convenience of once-monthly dosing. Based on previous studies [19, 20], we hypothesized that the effect of ibandronate in T2DM patients is not drastically different than in non-diabetic patients.

This study was conducted to provide evidence of the efficacy and safety of once-monthly ibandronate, at a 150 mg dose, in women with post-menopausal osteoporosis and T2DM. In addition, the influence of ibandronate on glucose metabolism was investigated in diabetic patients.

## Methods

### Study design

This study was a multicenter, prospective clinical trial with an open-label design. Patients were enrolled from Samsung Medical Center, Yeouido St. Mary’s Hospital, or Seoul National University Bundang Hospital, between 2018 and 2020. The study protocol was reviewed and approved by the institutional review board of Samsung Medical Center (SMC 2018–02-054), Yeouido St.Mary’s Hospital (SC18MEDV0024), and Seoul National University Bundang Hospital (B-1712/439–003). The study was performed in accordance with Declaration of Helsinki, and informed consent was obtained from all subjects. The current study was a prospective open-label clinical trial, and the protocol was submitted World Health Organization International Clinical Trial Registry Platform (NCT number: NCT05266261, Date of registration: 4 March 2022).

### Participants

The patients recruited were as those who had previously visited the hospital for other diseases, and who consented to the clinical trial explanation from the attending physician. Patients were enrolled based on the following inclusion criteria: a) age of at least 55 years at the time of screening; b) postmenopausal woman, defined as the absence of menstruation for at least 12 consecutive months; and c) a diagnosis of osteoporosis (indicated by a BMD T-score ≤ − 2.5 points in the lumbar spine, total hip, or femoral neck). Exclusion criteria were as follows: a) a history of osteoporosis treatment within 3 years of the study, b) underlying disease (e.g., heart failure, liver disease, renal disease, or malignancy) and/or the use of drugs that affect bone metabolism (e.g., steroids, immunosuppressants, gonadotropin-releasing hormone agonists, aromatase inhibitors, thiazolidinedione drugs, anticonvulsants, and antidepressants); and c) a history of adverse effects of bisphosphonate or difficulty taking the drug due to an inability to sit or the presence of upper gastrointestinal disease. Criteria for stopping the clinical trial were as follows: a) withdrawal of consent from study subjects, b) serious adverse reaction confirmed by the investigators c) violation of the protocol; or d) non-cooperation of subjects.

### Interventions

One tablet (150 mg of ibandronate + 24,000 IU of cholecalciferol) was provided on the same date each month with a sufficient amount of water at least 1 hour before the ingestion of food and other drugs. Calcium and vitamin D replacements were administered for all subjects during the study period (daily oral formulation: 1250 mg of calcium carbonate + 1000 IU of cholecalciferol). Study activities were discontinued if the subject withdrew consent or reported an adverse reaction to the study drug.

### Measurements

Subjects visited the clinic every 6 months and were instructed to fast for more than 8 hours in the morning of the visit day; blood pressure, pulse rate, respiration rate, and body temperature were measured at rest, and blood sampling was performed. BMD was measured at different centers using two types of dual-energy X-ray absorptiometry devices, either the Lunar Prodigy Advance (GE Healthcare., Chicago, IL, USA) or Hologic Delphi W (Hologic Inc., Marlborough, MA, USA), and was evaluated at baseline and at the end of the study. Harmonizing equations [21] were used to compare the baseline BMD values of the study groups. The trabecular bone score (TBS) of the lumbar spine was evaluated in the patient group that used the Lunar scanner. Serum C-terminal telopeptide of type I collagen (CTx) and procollagen type 1 N-terminal propeptide (P1NP) levels were measured by immunoassay methods using Elecsys kits – 07296355001 V4 and 07296509001 V4 (Roche Diagnostic Corp., Basel, Switzerland).

### Outcomes

The primary efficacy endpoint was the percentage change of BMD at the lumbar spine, femur neck, or total hip after 1 year, and secondary efficacy endpoints were percentage changes in the serum bone turnover markers CTx and P1NP. The primary safety endpoint was an adverse event requiring drug discontinuation; secondary safety endpoints were changes in fasting glucose or glycosylated hemoglobin (HbA1c) level at the start or end of the study in diabetic patients.

### Sample size

When the percentage change in lumbar spine BMD was compared between diabetic and non-diabetic patients, a difference of at least 2.0 percentage points was considered clinically meaningful, with consideration of error. The standard deviation was assumed to be 4.5 based on a previous study, and initial study planning calculated that about 45.5 study subjects were required for each group to achieve results with 80% power and at a 5% significance level (two-tailed test). Considering the anticipated dropout rate of 20%, a total of 120 patient enrollments were planned.

### Statistical analysis

Data normality was assessed through the Shapiro-Wilk Test. For continuous variables that formed normal distributions, the mean and standard deviation values were expressed and compared using a t-test. For continuous variables that did not form normal distributions, the median and interquartile ranges (IQR) were expressed and compared with the Wilcoxon rank-sum test. To analyze the percentage difference in BMD change, an analysis of covariance was performed with age as a covariate, and the result was expressed as the least square mean and its 95% confidence interval. Changes in bone turnover markers were analyzed using the generalized estimating equation for repeated-measures analysis and compared using Tukey’s method for multiple comparisons. Two sided *P* values less than 0.05 were considered statistically significant. Statistical analysis was performed using R version 4.1.0 (R Foundation for Statistical Computing, Vienna, Austria).

## Results

### Baseline characteristics

**Fig. 1:**
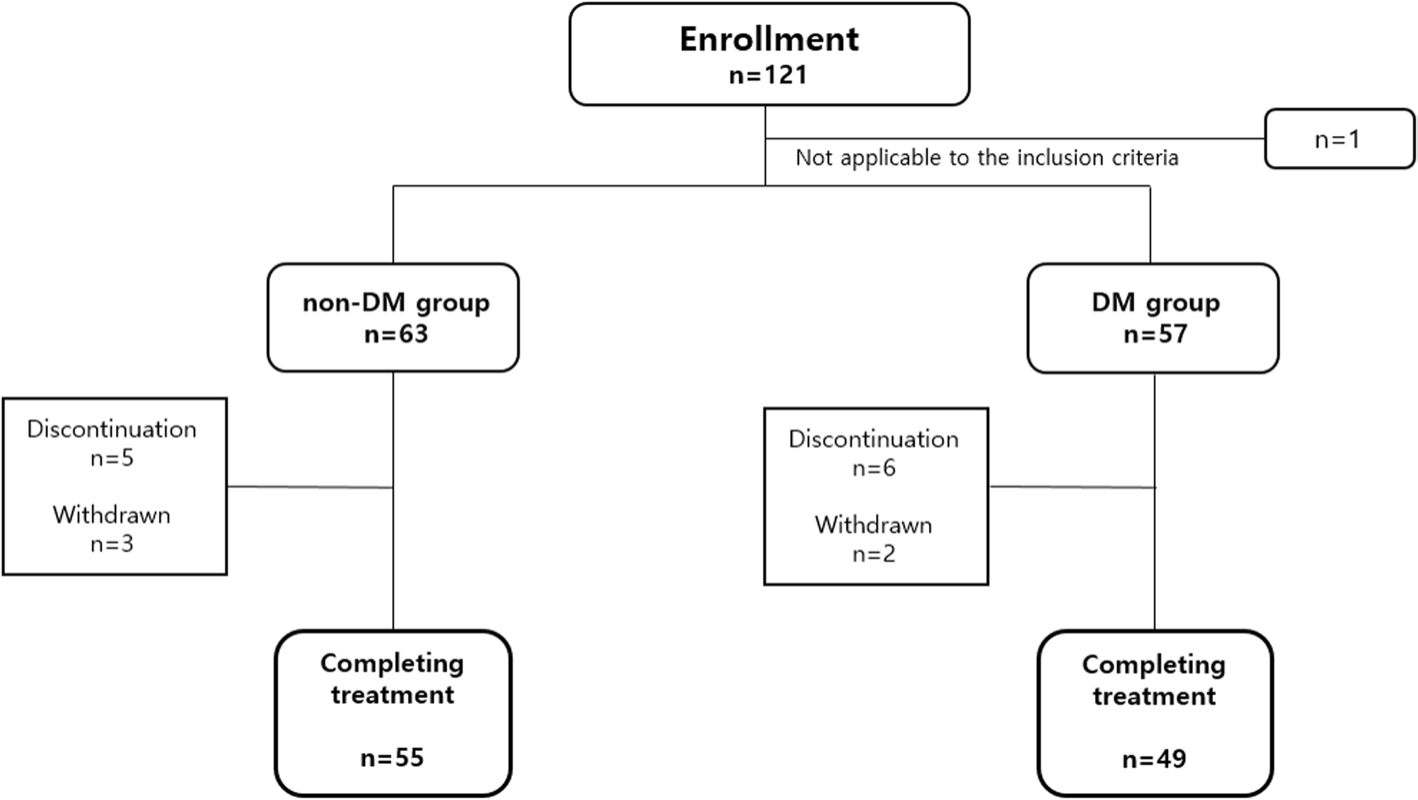
Patient follow-up

Among the 120 study participants, 104 (86.7%) completed the study (Fig. 1), and group analysis indicated that the diabetic group was older and had higher body mass index (BMI) values. However, BMD, TBS ​​and T-scores were not significantly different between the two groups, and there were no significant differences in baseline bone turnover markers or serum vitamin D level between the two groups (Table 1). The duration of T2DM in the diabetic group was a median of 12 years (IQR, 2–17.25 years). Treatment methods for T2DM varied from mono-drug therapy to insulin use. The baseline HbA1c level for T2DM patients ranged from 5.5 to 9.1% (Additional File 1: Fig. S1).

**Table 1:** Baseline characteristics according to group (per protocol)

	Non-DM (*n* = 55)	DM (*n* = 49)	P ^b^
Age, years (median (IQR))	66 (63–73)	73 (67–79)	< 0.001
Age at menopause	51 (48–53)	52 (48–54)	0.423
Menopause duration	16 (12–21)	23 (14–28)	0.037
Body-mass index, kg/m^2^ (mean (SD))	22.3 (2.6)	24.6 (2.6)	< 0.001
Fasting glucose, mg/dL (median, (IQR))	97 (94–106)	126 (113–149)	< 0.001
HbA1c, % (median, (IQR))	5.4 (5.3–.5.6)	6.4 (6.0–6.8)	< 0.001
Bone mineral density, g/cm^2^ (mean (SD))			
Lumbar	0.773 (0.078)	0.804 (0.709)	0.098
Femoral neck	0.656 (0.101)	0.671 (0.099)	0.663
Total hip	0.746 (0.099)	0.739 (0.106)	0.607
Bone mineral density, T score (mean (SD))			
Lumbar	−2.74 (0.75)	−2.54 (0.76)	0.180
Femoral neck	−2.08 (0.65)	−2.16 (0.77)	0.544
Total hip	−1.64 (0.75)	−1.91 (0.83)	0.085
Trabecular bone score ^a^ (mean (SD))	1.300 (0.058)	1.289 (0.076)	0.294
Previous fracture – number (%)	6 (10.9)	13 (26.5)	0.071
Serum β-CTX, ng/liter (median (IQR))	0.395 (0.260–0.593)	0.350 (0.225–0.503)	0.421
Serum P1NP, ng/liter (median (IQR))	53.1 (33.7–63.9)	45.4 (29.7–60.6)	0.346
25-Hydroxivitamin D, ng/mL (mean (SD))	25.57 (14.69)	31.36 (12.16)	0.192

^a^ Trabecular bone score values were available for 78 patients. ^b^ For continuous variables that formed normal distributions, the mean and standard deviation values were expressed and compared using a t-test. For continuous variables that did not form normal distributions, the median and interquartile ranges were expressed and compared with the Wilcoxon rank-sum test. Two sided *P* values less than 0.05 were considered statistically significant

### Efficacy

**Fig. 2:**
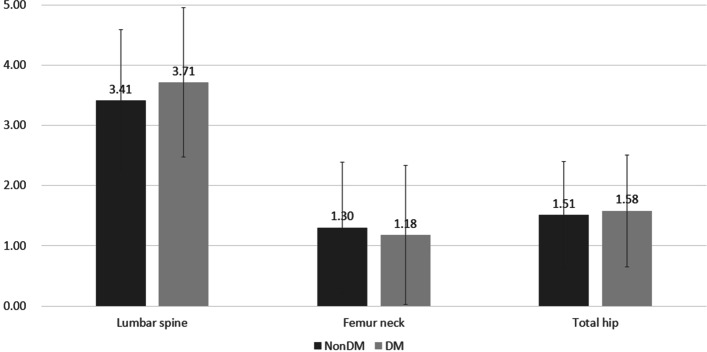
Percentage change in dual-energy X-ray absorptiometry (DXA) bone mineral density (BMD) at the lumbar spine, total hip, and femoral neck, presented as least square mean and 95% confidence interval. (*n* = 120, biologically independent samples; 63 for nondiabetes and 57 for diabetes)

Following 1 year of treatment, the BMD increased by 3.5% in the lumbar spine, 1.2% in the femur neck, and 1.5% in the total hip area for all treated patients. When the increments in BMD were investigated for each group, the results were 3.41% vs. 3.71% for the lumbar spine, 1.30% vs. 1.18% for the femur neck, and 1.51% vs. 1.58% in the total hip in the non-diabetes and diabetes group, respectively. There was no significant difference in these BMD changes between groups (Fig. 2). Changes in TBS values of the lumbar spine were analyzed for patients who had undergone imaging with the Lunar scanner. The mean (standard deviation) TBS values were 1.310 (0.058) at baseline and 1.333 (0.071) at the end of the study in the non-diabetic group and 1.289 (0.076) and 1.288 (0.088) in the diabetic group, respectively. Unlike BMD, no significant change in the TBS between before and after treatment was observed (*p* = 0.20 and *p* = 0.80).

**Fig. 3:**
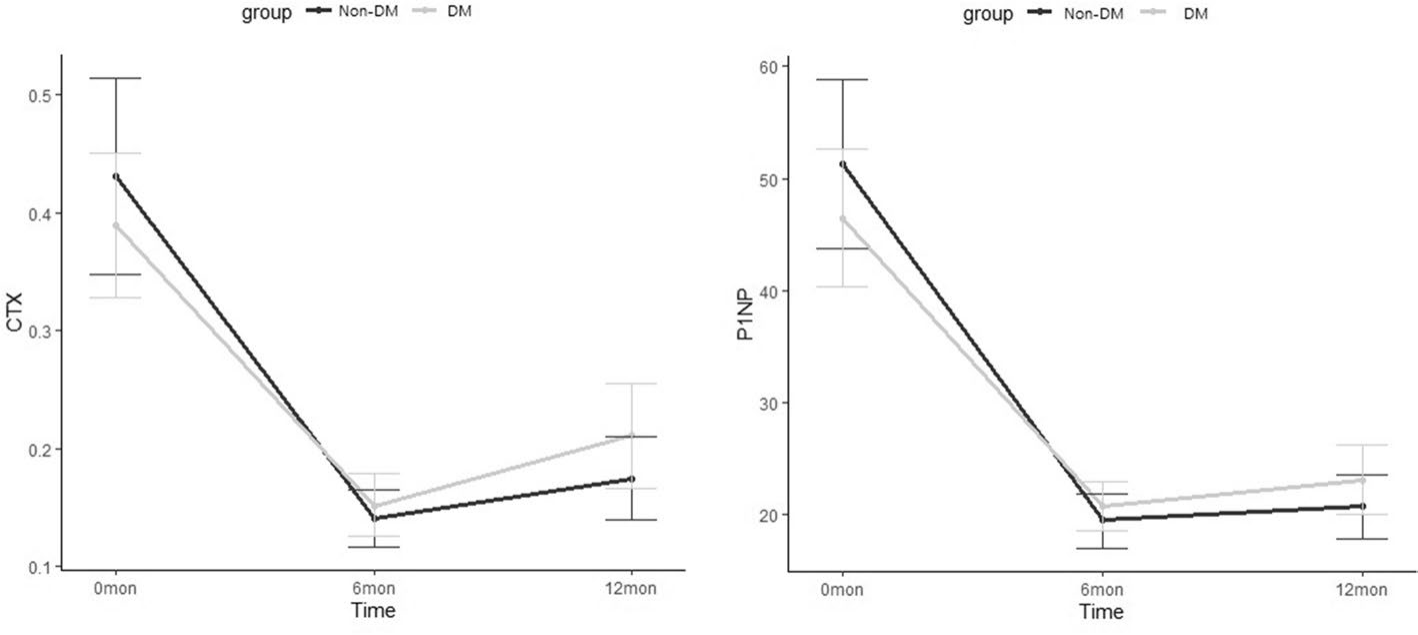
Percentage change in bone turnover markers presented as adjusted mean and 95% confidence interval using the generalized estimating equations for repeated measures analysis. (*n* = 76, biologically independent samples; 27 for nondiabetes and 49 for diabetes)

There was no difference in baseline level for changes in bone turnover markers between groups (Fig. 3). The levels of bone turnover markers decreased significantly following treatment (*p* < 0.001). The degrees of reduction at 6 months in the non-diabetes and diabetes groups were 58.1% vs. 48.6% (*p* = 0.09) for CTx and 54.9% vs. 51.1% (*p* = 0.19) for P1NP, respectively. In the diabetic group, the efficacy of ibandronate on BMD and bone turnover markers were evaluated according to the duration of diabetes, treatment method, and baseline HbA1c of the patient, but no significant difference was observed (Additional File 2: Table S1).

The number of patients with previously symptomatic compression fractures reported at the baseline screening visit was six in the non-diabetic group and 13 in the diabetic group, respectively. More patients in the diabetic group tended to report osteoporotic fractures, but this difference was not statistically significant. Radiologically confirmed lumbar compression fractures were investigated through baseline X-ray imaging, and nine and 15 patients in the non-diabetic group and the diabetic group, respectively, were identified as having such fractures; this difference was not statistically significant. Newly developed fractures were not analyzed as a study endpoint because the follow-up treatment period was limited to 1 year. However, no patients reported symptomatic fractures, including atypical fractures, during the study period. During follow-up, one radiologic compression fracture of the lumbar spine (L2) was confirmed in a patient in the diabetic group.

### Safety

A total of 11 adverse events (9.2%) occurred among the 120 included patients (Table 2). One patient in the non-diabetic group developed a persistent fever and was withdrawn from the study based on the investigator’s decision. Two patients in the non-diabetic group and three patients in the diabetic group stopped treatment due to myalgia. One patient in each group requested to discontinue the study due to dyspepsia, and one patient in the non-diabetic group and two patients in the diabetic group requested to discontinue the study due to weight loss. All adverse reactions recovered without sequelae after discontinuation of the drug. There was no significant difference in the frequency of adverse events between the two groups (*p* = 0.862). To evaluate the effects of ibandronate on glucose metabolism, levels of fasting glucose and HbA1c were evaluated in the diabetic group, and the differences between before and after treatment were not statistically significant (Table 3).

**Table 2:** Safety assessment based on treatment-emergent adverse event profiles (Intention to Treat)

	Non-DM (*n* = 63)	DM (*n* = 57)
Number of patients	5 (7.9%)	6 (10.5%)
Fever	1	0
Myalgia	2	3
Dyspepsia	1	1
Weight loss	1	2

*DM *diabetes mellitus

**Table 3:** Changes in glucose metabolism before and after treatment in the diabetic group

Clinical variables	Pre-treatment Median (Interquartile range)	Post-treatment Median (Interquartile range)	P
Fasting glucose, mg/dL(median, (IQR))	126 (112–142)	132 (116–146)	0.463
HbA1c, % (median, (IQR))	6.4 (6.03–6.80)	6.50 (6.03–7.07)	0.425

^a^ Two sided P values were calculated by the Wilcoxon rank-sum test

## Discussion

The purpose of this study was to determine whether monthly oral ibandronate therapy is as effective for retaining BMD in diabetic patients compared to non-diabetic patients. No significant difference was observed in the changes in BMD and suppression of bone turnover markers between the diabetic and non-diabetic groups. In addition, measurements of fasting glucose and HbA1c for evaluating glucose metabolism in diabetic patients did not significantly differ between before and after treatment.

In this study population, although the T2DM patient group was older, the baseline BMD and T-score values were not statistically different between the two groups. Therefore, patient age was corrected for analysis of covariance to compare differences in BMD changes. The average increase in lumbar spine BMD was 3.5% in all patients in this study, and there were increases of 3.4 and 3.7% in BMD in the lumbar spine in the non-diabetic and diabetic groups, respectively (Fig. 2). The observed 3.5% increase in BMD was lower than that reported in Western studies, but this result is similar to findings in a meta-analysis of randomized controlled trials [22]. There was also no significant difference in BMD change between the two groups in the femoral neck or the total hip area, which showed an increase less than 2%. However, the effect of ibandronate on lumbar spine BMD was confirmed, which is consistent with the results of a previous large-scale study that documented significant prevention of vertebral fracture with ibandronate therapy [23].

Discordance has been reported in that patients with T2DM have a high BMD but are at an increased fracture risk [24, 25]. Previous investigators have reported significantly lower TBS values in T2DM patients, and thus attempted to explain the increased fracture risk in diabetic patients through the mechanism of low TBS values [26, 27]. However, we did not observe any significant differences in baseline TBS values or the degree of change between before and after one year of ibandronate treatment in either group in this study. This is consistent with previous observations that changes in TBS were less responsive to bisphosphonate treatment compared to BMD [28, 29]. The coefficient of variation indicating reproducibility or precision of TBS is more than twice that of BMD [30], and it is thought that the number of patients confirming statistical significance is greater than the sample size of this study. Therefore, the interpretation of TBS in T2DM patients may be controversial, and further studies are needed.

Similar to the results of previous reports [31, 32], the degree of biomarker reduction was about 50% for both CTx and P1NP in this study. There was no significant difference in baseline biomarker levels in the two groups, and the effect could be verified by the decrease in bone turnover markers without a significant difference between the diabetic and non-diabetic groups (Fig. 3). Diabetic patients generally tended to exhibit low bone turnover [33, 34], but bone turnover markers were not significantly different in this study. This may be due to the fact that most patients had good glycemic control with a HbA1c level under 7%. However, considering that the diabetic group was older than the non-diabetic group, it is a meaningful that the changes in bone turnover markers were not significantly different in this study. Low bone turnover in diabetic patients can be related to side effects associated with the use of bisphosphonates. However, the inhibition of bone turnover in the diabetic group was similar to that in the non-diabetic group according to the results of this study. In addition, similar changes in bone turnover markers in both groups prove that it is justified to use bone turnover markers to monitor drug efficacy even in diabetic patients.

In terms of safety, the proportion of patients who discontinued study participation was almost the same in both groups, and there was no significant difference in the incidence of adverse effects between the groups (Table 2). Although the diabetic group was older and more side effects can be expected in this population due to polypharmacy [35], the absence of a difference in the incidence of side effects in the groups demonstrates the safety of the drug. Therefore, we suggest that monthly ibandronate (150 mg ibandronate + 24,000 IU vitamin D tablet) is tolerable in postmenopausal women with diabetes.

Bisphosphonate therapy reduced bone turn-over and resulted in lowering osteocalcin secretion, which may influence glucose metabolism. However, it is not clear how bisphosphonate treatment affects glucose metabolism in diabetic patients. Researchers previously reported that the effect of altered osteocalcin levels on glucose metabolism with bisphosphonate therapy was inconsequential [36, 37]. Some studies insist a rather favorable effect of bisphosphonate therapy in diabetic patients [38, 39]. The results of this study also show no differences in fasting glucose and HbA1c levels between before and after treatment (Table 3). Based on the results of our study, the effect of ibandronate on diabetes is not significant, so the use of ibandronate in T2DM patients appears to be safe in terms of glucose metabolism. Subgroup analysis was performed to further assess the diabetic group of this study based on patient distribution (Additional File 1: Fig. S1) and treatment effects (Additional File 2: Table S1). However, no significant difference was noted in the efficacy of ibandronate according to diabetic status.

This study had several limitations. First, researcher intervention cannot be excluded since the study was conducted with an open-label design. In particular, the recruitment process of patients in this study was conducted with those who received research proposals from the attending physician, rather than open recruitment. Second, the number of subjects in this study was smaller than in the previous study [40], and the research approach might have overlooked an effect on femur neck and total hip BMD, where the effect of ibandronate was relatively small. In addition, a larger number of patients is needed to investigate TBS change due to precision error. Third, patients in the diabetes group were composed of patients with good glycemic control and different diabetic treatment methods, it is difficult to confirm definite influence of ibandronate on glucose metabolism from this study alone. Therefore, additional studies are necessary for uncontrolled diabetes mellitus or type 1 diabetic patients. Finally, it is difficult to evaluate the effect of ibandronate treatment on long-term fracture prevention based only on the results of this study because of the one-year study duration. Nevertheless, this study is a rare study designed to investigate the difference in treatment response to osteoporosis medications between patients with and without diabetes, and the results provide data that support the use of ibandronate therapy in patients with diabetes. To the best of our knowledge, this study is the first study on the use of ibandronate in diabetic patients.

## Conclusions

In conclusion, bisphosphonate therapy showed similar increases in BMD and decreases in bone turnover markers in postmenopausal women with and without diabetes. Monthly oral ibandronate can be a safe and effective therapeutic option in postmenopausal osteoporosis patients with T2DM.

## Supplementary Information

**Additional file 1.**

**Additional file 2.**

## Data Availability

The data that support the findings of this study are available from the corresponding author upon reasonable request.

## Abbreviations

*T2DM*: Type 2 diabetes mellitus
*BMD*: Bone mineral density
*DEXA*: Dual-energy X-ray absorptiometry
*TBS*: Trabecular bone score
*CTx*: C-terminal telopeptide of type I collagen
*P1NP*: Procollagen type 1 N-terminal propeptide
*HbA1c*: Glycosylated hemoglobin
*IQR*: Interquartile ranges
*BMI*: Body mass index
