# Supplementary material for: Efficacy of bisphosphonate therapy on postmenopausal osteoporotic women with and without diabetes: a prospective trial
Source: BMC Endocr Disord. 2022 Apr 11;22:99. doi: 10.1186/s12902-022-01010-w (PMC9004203; doi:10.1186/s12902-022-01010-w)
Supplement: Supplementary file 1 — Additional file 1. [file 12902_2022_1010_MOESM1_ESM.docx]

**
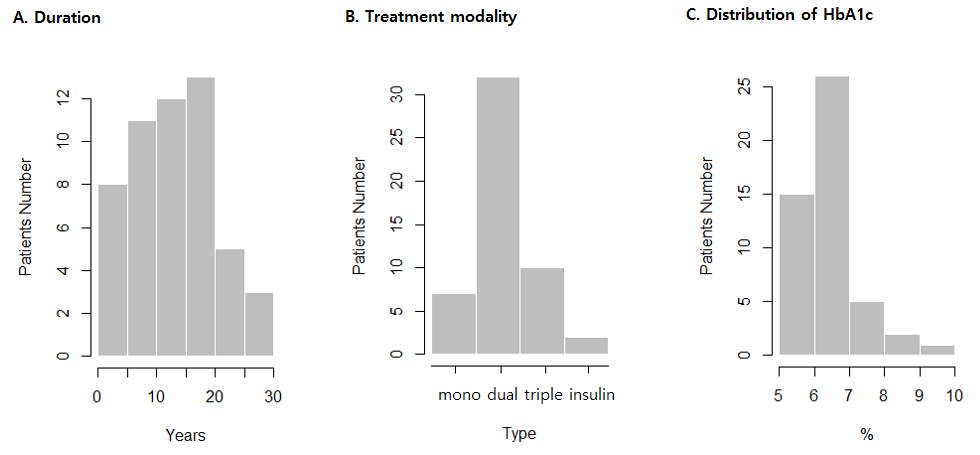
**

**Supplementary Figure 1.** Baseline characteristics of the type 2 diabetes mellitus group. HbA1c, glycosylated haemoglobin.
