# Supplementary material for: Efficacy of bisphosphonate therapy on postmenopausal osteoporotic women with and without diabetes: a prospective trial
Source: BMC Endocr Disord. 2022 Apr 11;22:99. doi: 10.1186/s12902-022-01010-w (PMC9004203; doi:10.1186/s12902-022-01010-w)
Supplement: Supplementary file 2 — Additional file 2. [file 12902_2022_1010_MOESM2_ESM.docx]

**Supplementary Table 1.** Therapeutic effects in the T2DM group according diabetic status.

| **T2DM status** | **Subgroups** | **BMD change,**  **L-spine (%)** | **BMD change,**  **femur neck (%)** | **BMD change,**  **Total hip (%)** | **Bone turnover,**  **CTx change (%)** | **Bone turnover,**  **P1NP change (%)** |
| --- | --- | --- | --- | --- | --- | --- |
| **Duration** | DM < 10 years (n=17) | 2.47 (-0.35-5.19) | 2.34 (-0.28-4.09) | 1.23 (0.24-2.17) | 62.3 (47.8-72.0) | 53.7 (34.3-62.8) |
|  | DM > 10 years (n=32) | 4.49 (1.97-6.54) | 0.67 (-1.69-2.94) | 0.92 (-0.17-2.39) | 56.7 (26.7-70.3) | 58.3 (37.1-68.0) |
|  | ***P*** ^a^ | 0.210 | 0.151 | 0.855 | 0.343 | 0.685 |
| **Treatment** | Mono or dual oral medications (n=37) | 4.49 (1.75-3.47) | 0.90 (-1.24-2.81) | 1.04 (0.00-2.04) | 57.1 (41.8-70.3) | 40.1 (30.7-59.0) |
|  | Sulfonylurea or insulin (n=12) | 2.36 (0.50-6.06) | 3.43 (-1.41-3.82) | 1.84 (0.28-3.66) | 58.8 (23.5-73.4) | 58.1 (34.4-67.8) |
|  | ***P*** ^a^ | 0.838 | 0.456 | 0.516 | 0.944 | 0.507 |
| **Control** | HbA1c < 7% (n=37) | 3.88 (1.00-5.39) | 1.41 (-1.23-3.72) | 1.04 (-0.12-3.43) | 56.3 (39.7-71.9) | 58.1 (35.2-68.0) |
|  | HbA1c > 7% (n=12) | 4.27 (1.97-6.06) | 1.00 (-1.98-3.06) | 1.37 (0.09-2.17) | 61.8 (39.3-70.6) | 42.7 (25.4-63.9) |
|  | ***P*** ^a^ | 0.541 | 0.336 | 0.707 | 0.699 | 0.337 |

T2DM, type 2 diabetes mellitus; BMD, bone mineral density; CTx, C-telopeptide of collagen type 1; P1NP, procollagen type 1 N-terminal propeptide; HbA1c, glycosylated haemoglobin. ^a^ Two sided P values were calculated by the Wilcoxon rank-sum test.
